# Herpetic Pseudotumor of the Nostril: A Report of Facial Herpes Vegetans in a Patient With Chronic Lymphocytic Leukemia

**DOI:** 10.7759/cureus.38702

**Published:** 2023-05-08

**Authors:** Cheri Frey, Jewell Dinkins, Stephen Suah, Kimberly Merkel

**Affiliations:** 1 Dermatology, Howard University College of Medicine, Washington, D.C., USA; 2 Dermatology, Meharry Medical College, Nashville, USA; 3 Dermatology, Foxhall Dermatology, Washington, D.C., USA; 4 Dermatology, University of Florida, Gainesville, USA

**Keywords:** herpes, facial herpes, herpetic pseudotumor, herpetic pseudotumor of the nostril, facial herpes vegetans, dermatology, pseudotumor, acyclovir, chronic lymphocytic leukemia (cll), herpes simplex virus type 1

## Abstract

Cutaneous herpes simplex virus (HSV) infections characteristically present with a vesicular eruption on an erythematous base that is easily recognized and diagnosed. Immunocompromised patients, such as those with HIV/AIDS or malignancy, may develop atypical verrucous lesions, necrotic ulcers, and/or erosive vegetative plaques. The most common location for these atypical lesions is the anogenital region. Few facial lesions have been reported in the literature. We report a case of a rapidly growing vegetative lesion on the nose of a 63-year-old male with chronic lymphocytic leukemia. A skin biopsy and immunostaining confirmed a diagnosis of herpes simplex. The patient was successfully treated with IV acyclovir.

Infection is the main cause of mortality among patients with chronic lymphocytic leukemia (CLL), and reactivation of herpes is common. Occasionally, HSV may present in an unusual manner and/or location, creating a diagnostic dilemma that can potentially delay diagnosis and treatment. The present report highlights the importance of considering atypical presentations of HSV in immunosuppressed patients, regardless of lesion location, as early detection and treatment are especially critical in this population.

## Introduction

Cutaneous herpes simplex virus (HSV) infections characteristically present with a vesicular eruption on an erythematous base that is easily recognized and diagnosed [1]. Immunocompromised patients, such as those with HIV/AIDS or malignancy, may develop atypical verrucous lesions, necrotic ulcers, and/or erosive vegetative plaques [2]. The most common location for these atypical lesions is the anogenital region. A few facial lesions have been reported in the literature.

## Case presentation

A 63-year-old Caucasian man with a past medical history of chronic lymphocytic leukemia (CLL), last treated with chlorambucil 12 months prior, presented to his oncology clinic with a three-week history of a rapidly growing, crusted nodule on his left nostril. The patient complained of lesion tenderness and difficulty breathing due to obstruction of the left nare. He reported the lesion began as a "pimple." He was treated with cephalexin, clindamycin, and bacitracin without improvement. The patient had no previous similar growths or a history of skin cancer.

**Figure 1:**
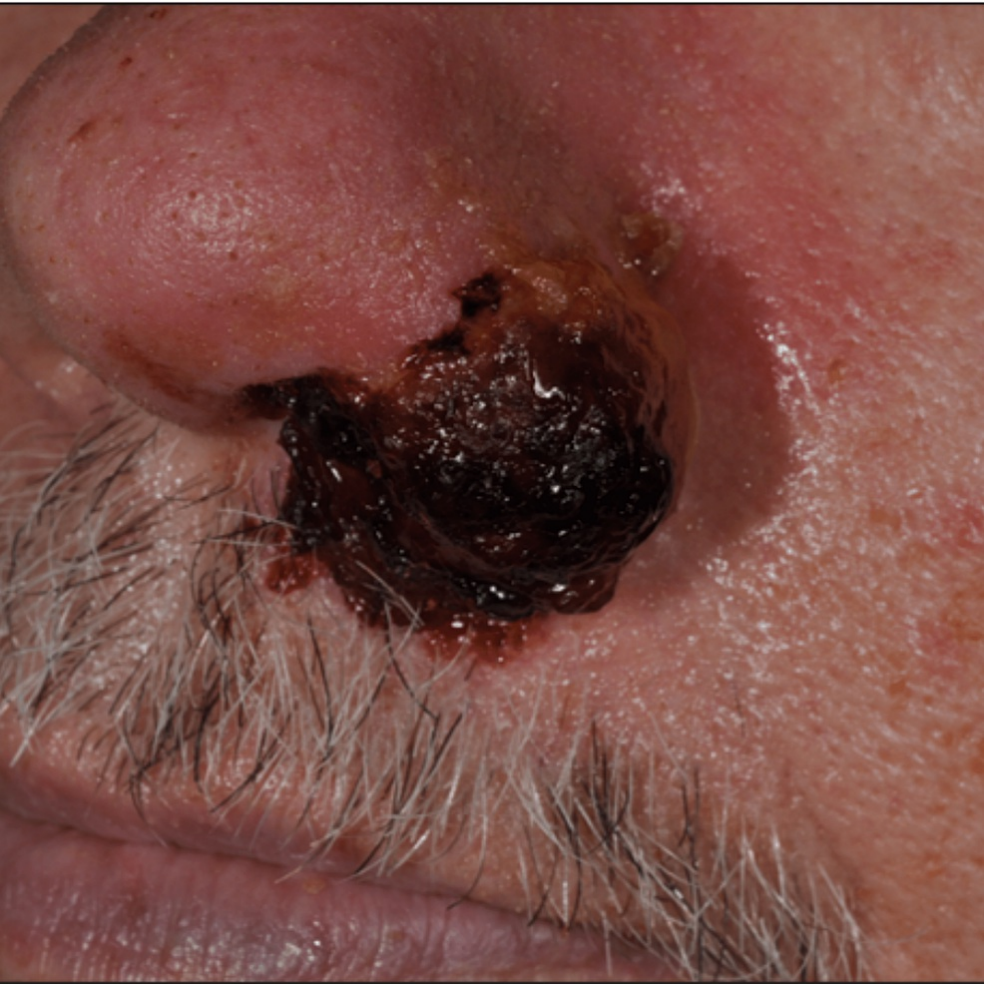
Herpes simplex virus-induced exophytic tumor of the left nasal ala and naris.

A physical exam revealed a large, partially necrotic, exophytic tumor involving the left nasal ala and nasal tip with obstruction of the left nostril (Figure 1).

**Figure 2:**
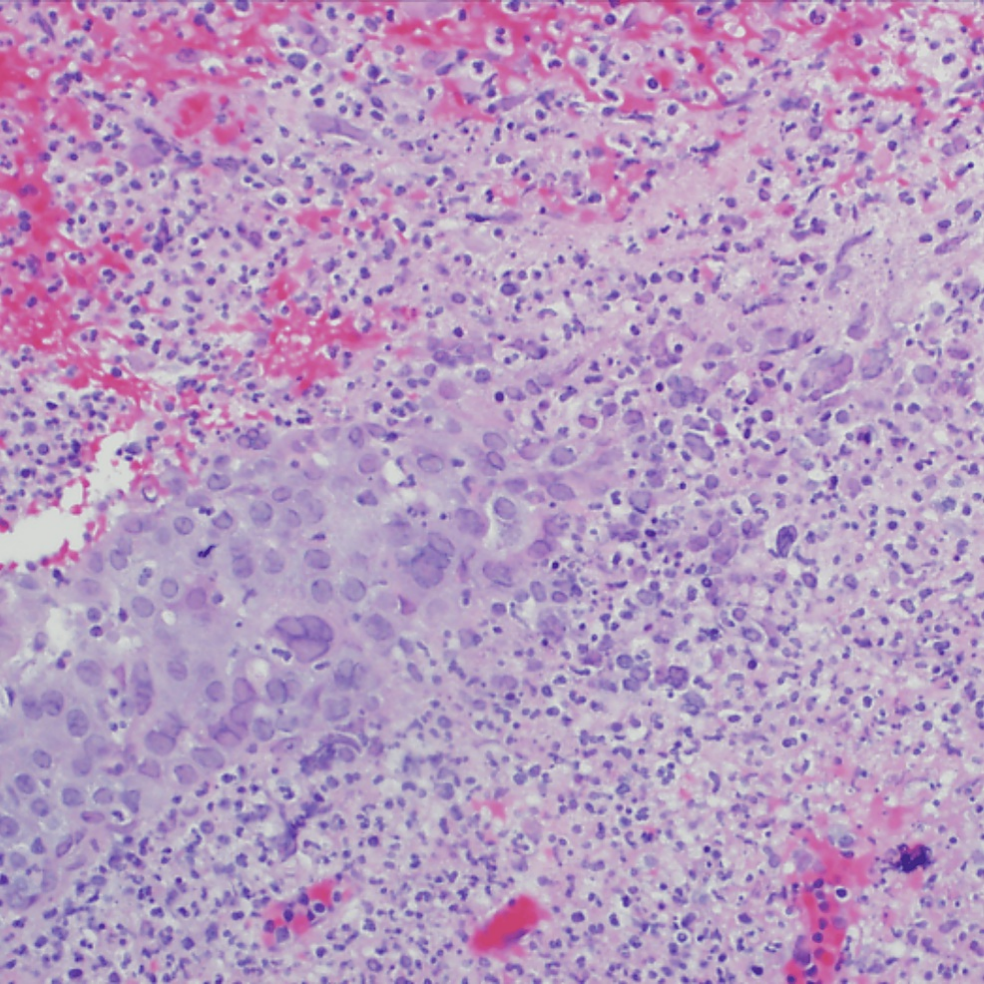
A high-power view demonstrates viral cytopathic changes, including multinucleated giant cells.

At that point, the clinical differential diagnosis included cutaneous lymphoma, squamous cell carcinoma, basal cell carcinoma, atypical mycobacterial infection, and invasive fungal infection. A scoop-shave biopsy was performed, and specimens were sent for pan-cultures. Pathology results revealed an ulcerative lesion as well as a dense dermal infiltrate of acute and chronic inflammation. The epidermis showed focal hyperplastic changes and an intraepidermal collection of acantholytic cells with giant cell formation, nuclear molding, and margination of nuclear chromatin (Figure 2).

Immunostains for HSV were positive, and a diagnosis of vegetative herpes simplex virus infection was made. Pan-culture results are not currently available. The patient was started on oral valacyclovir (1g three times daily), which resulted in suboptimal clinical improvement. He was then switched to intravenous acyclovir 5-10 mg/kg (every eight hours for seven days) and showed marked clinical improvement. He was subsequently referred to the infectious disease unit for further evaluation. It is unknown if he was placed on suppression therapy.

## Discussion

The most characteristic presentation of cutaneous herpes simplex infection is that of clustered vesicles on an erythematous base or grouped ulcerations with scalloped borders. Atypical forms may present as nodular lesions or mimics of condyloma [3-4]. Vegetative herpes simplex, also known as herpes vegetans and hypertrophic herpes, is an atypical variant of cutaneous herpes simplex typically found in a genitocrural location. To our knowledge, there has only been one other case of a vegetative herpetic lesion within the nostril reported in the literature and only three cases of vegetative herpetic lesions on the face.

Vegetative HSV has been most commonly associated with HIV/AIDS patients but may also appear in patients with malignancy or those receiving immunosuppressive therapy [5-6]. Reactivation of the herpes virus in patients with CLL arises from functional immune suppression due to hypogammaglobulinemia, dysfunctional leukemic B cells, cytokines, and aberrant T cell activation [7-8]. While the classic presentation of HSV is easily diagnosed based on clinical features, atypical lesions require a high degree of clinical suspicion. Additional testing should include HSV polymerase chain reaction (PCR) tests, direct fluorescent antibody testing, viral culture, and/or skin biopsy. Khera et al. reported a case of cutaneous HSV misdiagnosed as acrodermatitis of Hallopeauand, and the delay in diagnosis led to the patient receiving intralesional steroid injections. Similarly, our patient was first treated with antibiotics [8].

Cutaneous HSV infections in patients with CLL can also present a therapeutic challenge [9]. An increased incidence of dissemination, recurrence, and resistance to acyclovir has been reported among patients with CLL and cutaneous HSV infections. In particular, our patient did not respond to oral valacyclovir and was transitioned to IV acyclovir. Although the patient’s lack of response was likely an issue with dose or blood levels, this highlights the importance of considering the possibility of acyclovir resistance for patients who do not respond to acyclovir within five to seven days.

## Conclusions

In conclusion, we present a case of a rapidly growing exophytic pseudo-tumor in a patient with CLL. Herpes vegetans should be considered in the differential for all immunocompromised patients with rapidly growing exophytic, tumor-like lesions. While vegetative HSV more commonly arises as genital and perianal lesions, it is important to remember that they can present on the face. Knowledge of this atypical cutaneous manifestation of HSV in the immunosuppressed patient population is critical for management and timely treatment. 
